# Effects of tilt and decentration of Visian Implantable Collamer Lens (ICL V4c) on visual quality: an observational study

**DOI:** 10.1186/s12886-022-02499-4

**Published:** 2022-07-05

**Authors:** Lingling Niu, Zhe Zhang, Huamao Miao, Jing Zhao, Meiyan Li, Ji C. He, Peijun Yao, Xingtao Zhou

**Affiliations:** 1 Department of Ophthalmology and Optometry, Eye & ENT Hospital, Fudan University, Shanghai, China; 2 NHC Key Laboratory of Myopia (Fudan University), Key Laboratory of Myopia, Chinese Academy of Medical Sciences, Shanghai, China; 3 Shanghai Research Center of Ophthalmology and Optometry, Shanghai, China; 4 New England College of Optometry, Boston, MA USA

**Keywords:** Implantable Collamer Lens, Myopia, Decentration, Tilt, Visual quality

## Abstract

**Background:**

The central hole of the Visian Implantable Collamer Lens (ICL V4c) provides a reference to observe its tilt or decentration. This study aimed to investigate the tilt and decentration effects of ICL V4c on visual quality after implantation.

**Methods:**

A total of 135 eyes from 69 patients who underwent ICL V4c implantation were included in this study. Evaluation of uncorrected distance visual acuity (UDVA), corrected distance visual acuity (CDVA), and aberrations were performed 6-months postoperatively. The anterior segment parameters were collected using CASIA2 anterior segment-optical coherence tomography, tilt and decentration of ICL V4c were analyzed using MATLAB software. All patients received questionnaires to investigate the visual quality postoperatively.

**Results:**

The safety and effectiveness were 1.18 ± 0.17 and 1.11 ± 0.18, respectively. No significant changes were observed regarding higher-order and spherical aberrations after the operation; however, coma and trefoil significantly increased compared to preoperative values. The average total decentration and tilt was 0.21 ± 0.12 mm and 2.54 ± 1.00°, respectively. Horizontal, vertical, and total values of tilt and decentration were not significantly associated with postoperative CDVA, UDVA, and aberrations. The most common visual symptom was halo, and 97.04% of patients had a satisfaction score ≥ 8. The total or horizontal tilt was significantly positively correlated with the frequency, severity, and bothersome scores from the questionnaires.

**Conclusions:**

ICL V4c implantation can obtain high visual quality and patient satisfaction. Although the degree of tilt and decentration after ICL V4c implantation was small, a positive effect on subjective visual quality was observed.

**Supplementary Information:**

The online version contains supplementary material available at 10.1186/s12886-022-02499-4.

## Background

In 2005, the United States Food and Drug Administration approved the use of the Implantable Collamer Lens (ICL, STAAR Surgical, Nidau, Switzerland), which has since demonstrated its safety and efficacy as a treatment method for high myopia [1–3]. The ICL is reversible, and maintains the central cornea integrity and promotes early recovery of visual acuity [4, 5]. The CentraFLOW technology [6] in the Visian Implantable Collamer Lens (ICL V4c) contains a 360-μm central hole that promotes aqueous humor circulation, which reduces the incidence of cataract formation, decreases reduction of corneal endothelial cells and eliminates the need to perform neodymium: YAG (Nd: YAG) iridotomy before ICL implantation [7–9].

Studies have shown that ICL V4c implantation produces better visual quality compared to that of laser-assisted in situ keratomileusis and small incision lenticule extraction. However, post-ICL implantation complications including high-order aberrations and coma aberrations increased still occur, as well as symptoms, including halo and glare [10–14].

Tilt and decentration of the intraocular lens (IOL) can cause higher-order aberrations, which may lead to deterioration of visual function [15, 16]. Therefore, ICL tilt and decentration effects on postoperative visual quality those patients that had no history of. The hole in the ICL provides a reference that allows observation of the position of the lens to evaluate the presence of tilt or decentration. The ICL hole is not usually located in the central pupil [17]; however, previous studies regarding the effect of ICL V4c decentration on visual acuity were in-vitro studies, and in-vivo experiments are lacking [18, 19]. The new generation anterior segment optical coherence tomography (AS-OCT) possesses a 1310-nm laser wavelength (CASIA2, Tomey, Japan), and has the advantages of high axial and transverse resolutions of 10 μm and 30 μm, respectively, high repeatability, and is non-mydriatic [20]. Furthermore, it can clearly display the anterior and posterior surfaces of the cornea, ICL, and crystalline lens. Additionally, AS-OCT automatically measures the tilt and decentration of the lens as a reference to the corneal topographic axis. This is the first study aimed at measuring and analyzing the post-implantation tilt and decentration of the ICL V4c and evaluating their the effects on patients’ visual quality.

## Methods

### Subjects

All patients were fully informed of the details and potential risks of the procedure, and wrote informed consent was obtained from all patients. The Ethics Committee of the Fudan University EENT Hospital Review Board approved the study protocol (No. 2016038), and the study adhered to the tenets of the Declaration of Helsinki.

In this observational study, we recruited individuals who underwent ICL V4c implantation at our hospital. The inclusion criteria were as follows: patients aged 20 – 42 years, those with a stable refractive error (≤ 0.50 D change per year in refractive error for the past 2 years), minimum anterior chamber depth (ACD) of 2.8 mm, a minimum endothelial cell density (ECD) of 2000 cells/mm^2^, and no contact lens use for at least 2 weeks. The exclusion criteria were as follows: patients with comorbid eye disorders, suspicion of keratoconus and presence of comorbid systemic diseases.

### Visian implantable collamer lens

The power calculation of the ICL V4c (STAAR Surgical, Nidau, Switzerland) was performed using a modified vertex formula based on the preoperative refractive parameters, according to the manufacturer’s instructions. The size of the implanted ICL V4c was determined from the white-to-white and ACD both obtained by pentacam.

### Surgical procedure

An experienced surgeon (XZ) performed all ICL V4c implantations, as described previously [21]. Briefly, pupils were dilated preoperatively. A mark was made to show the horizontal axis on the limbus to allow the use of a Toric ICL (TICL). Using an injector cartridge, the ICL was implanted via a 3.0-mm temporal corneal incision. Then a moderate viscoelastic surgical agent (1% sodium hyaluronate) was injected into the anterior chamber, and the ICL V4c lens was placed and positioned in the posterior chamber. The viscoelastic surgical agent was washed away entirely using a balanced salt solution, and a miotic agent was instilled. Postoperative medications included antibiotic, non-steroidal anti-inflammatory, steroidal, and artificial tears eye drops.

### Follow-up examination

All patients underwent preoperative and postoperative ocular examinations. The following main parameters were evaluated: uncorrected distance visual acuity (UDVA), corrected distance visual acuity (CDVA), subjective manifest refraction, intraocular pressure (IOP, Canon, Japan), corneal topography and vault (Pentacam HR, Oculus Optikgeräte GmbH, Wetzlar, Germany), wavefront aberration analysis (WASCA Wavefront Analyzer, Carl Zeiss Meditec, Germany), ECD (SP-95 2000P, Topcon Corporation, Japan), and ultrasound biomicroscopy (Quantel Medical, French). Patients were followed-up after 6 months postoperatively. The wavefront aberration was analyzed using the Zernike polynomials, recommended by the Optic Society of America, under an analysis of 5 mm pupil diameter.

### AS-OCT measurement

The ocular anterior segment parameters were measured using AS-OCT (CASIA2; TOMEY, Nagoya, Japan). The chin and forehead of patient was fixed and gazed at a fixation target to fully expose the corneal limbus. A total of 128 anterior segment tomographic images of the entire circumference were obtained in only 2.4 s, and the measurements were repeated three times by the same examiner. Each OCT image clearly presents the anterior and posterior surfaces of the cornea, lens, and ICL. Sixteen images from 0° to 180° with different directions (0, 11, 23, 34, 45, 56, 68, 79, 90, 101, 113, 124, 135, 146, 158, and 169 degrees) were selected for each eye for analysis.

### Tilt and decentration values

Detailed description of the measurements of tile and decentration of the ICL is presented in the Supplementary Method. Briefly, the raw measurement images of AS-OCT were exported to MATLAB software (R2018a, The MathWorks, Inc., Natick, MA, USA) with a purpose-designed program. Four registration lines were manually adjusted to align the anterior and posterior corneal surfaces and ICL surfaces. Then the location of marked point on the image were expressed in pixels (x and y, per pixel equaled to 7.749 μm) relevant to the coordinate XY axis. The corneal topography axis was defined as the connecting line of the fixation point of the machine and the corneal vertex, which was vertical to the X axis in each image. The tilt value of ICL was determined by averaging the degrees of rotation of the registration lines fitted to the anterior and posterior surfaces of the ICL in each image. The highest value of tilt within the 16 images represented the total tilt value. The horizontal and vertical tilt were the values on the 0- and the 90-degree images.

The decentration was defined as the horizontal distance between the center of the central hole and the corneal topographic axis. Since the location of the ICL central hole was not present in all 16 images, the highest value of decentration among the images with a central hole represented the total decentration value. The horizontal and vertical decentration values were based on the total decentration value and calculated according to the image direction and the Pythagorean theorem.

Only absolute values of tile and decentration were used for analysis. All image analyses were performed independently by two examiners and the consistency of the results were evaluated.

### Questionnaire design

All patients completed a questionnaire to report their subjective quality of vision during the follow-up at 6 months postoperatively. The Quality of Vision (QoV) Questionnaire by McAlinden consists of a Rasch-tested, linear-scaled, 30-item instrument that includes three scales and provides a QoV score in terms of symptom frequency, severity, and bothersome [22]. It is suitable for measuring QoV in patients with refractive correction. The 10 symptoms included glare, haloes, starbursts, hazy vision, blurred vision, distortion, double vision, fluctuation, focusing difficulties, and depth perception. Patients were asked regarding their overall satisfaction with the surgery, with “0” being dissatisfied and “10” being a perfect score of satisfied.

### Statistical analyses

Statistical analyses were performed using R software Version 3.4.3 (http://www.R-project.org). Continuous and categorical variables were expressed as means ± standard deviations and frequencies (percentages), respectively. A paired t-test was used to analyze the difference between postoperative horizontal and vertical tilt or decentration. The generalized estimating equation model was used to analyze the correlation between tilt or decentration and aberration or visual quality score, which was adjusted for age, eye, preoperative refraction, and preoperative scotopic pupil at treatment. Statistical significance was set at *p* < 0.05.

## Results

### Subjects’ biometrics

A total of 135 eyes from 69 patients (56 women and 13 men) with an average age of 28.88 ± 5.46 years (20–40 years) were enrolled in this observational clinical study; among them, 89 eyes were implanted with a TICL. The preoperative mean CDVA was 1.01 ± 0.17 (0.5 to 1.2) and the mean spherical equivalent (SE) was -10.16 ± 2.99 diopters (D) (-4.0 to -18.50 D). At the 6-month follow-up, the mean postoperative UDVA and CDVA were 1.12 ± 0.23 (0.5 to 1.5) and 1.18 ± 0.19 (0.6 to 1.5), respectively; the mean postoperative SE was -0.13 ± 0.44 (-2.25, 0.75). The efficacy index (postoperative UDVA/preoperative CDVA) was 1.11 ± 0.18 and the safety index (postoperative CDVA/preoperative CDVA) was 1.18 ± 0.17. All 135 eyes achieved a postoperative SE within ± 1.0 D of the attempted SE.

No significant changes in IOP were observed between the preoperative (15.25 ± 2.60 mmHg) and 6 months postoperative (14.87 ± 2.82 mmHg) values (*p* = 0.61). The number of ECD decreased by 0.6% compared with the preoperative ECD (2577.71 ± 214.58 cell/mm^2^ vs. 2568.66 ± 229.75 cell/mm^2^, *p* = 0.61). The vault was 554.62 ± 227.83 μm (165–1360 μm), and no clinical cataracts were observed. Additionally, 91.9% of patients had a scotopic pupil with a preoperative diameter > 5.8 mm.

### Tilt and decentration

The detailed results of the total, horizontal, and vertical tilt and decentration of all ICLs at 6 months postoperatively are presented in Table 1.

**Table 1:** The tilt and decentration of ICL V4c postoperatively (*N* = 135 eyes)

Characteristic	Mean ± SD	Range (Minimum, Maximum)
Total tilt (°)	2.54 ± 1.00	0.35, 4.53
Horizontal tilt (°)	1.78 ± 1.06	0.05, 4.65
Vertical tilt (°)	1.22 ± 0.91	0, 3.85
Total decentration (mm)	0.21 ± 0.12	0.019, 0.520
Horizontal decentration (mm)	0.14 ± 0.10	0, 0.375
Vertical decentration (mm)	0.14 ± 0.10	0, 0.442

*ICL V4c* Visian Implantable Collamer Lens, *SD*  Standard deviation

**Fig. 1:**
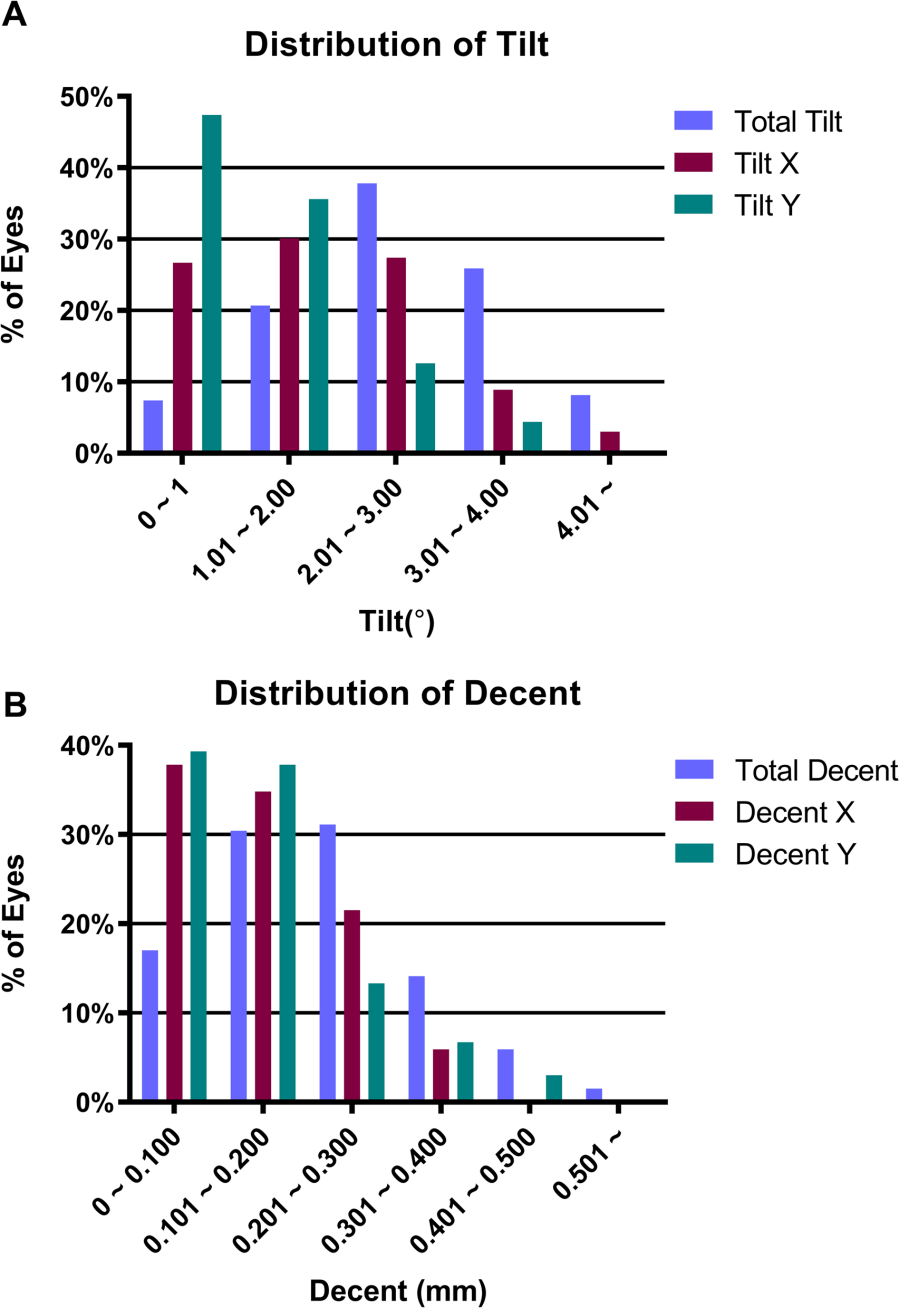
**A** Percentage of ICL V4c tilt at the six-month follow-up; **B** Percentage of ICL V4c decentration at the six-month follow-up

The horizontal tilt was significantly greater than the vertical tilt (*p* = 0.000). Among them, 28.1% of the operated eyes were tilted within 2.0°, 91.85% within 4.0°, and the maximum did not exceed 5.0° (Fig. 1A). No significant difference was observed between the horizontal and vertical decentration (*p* = 0.730). The distribution of the decentration was 47.4% within 0.2 mm, 98.5% within 0.5 mm, and the maximum decentration value did not exceed 0.6 mm (Fig. 1B).

### Aberration

At the 6-month follow-up, both coma and trefoil were significantly increased, from 0.12 ± 0.09 μm and 0.09 ± 0.08 μm to 0.14 ± 0.09 μm (*p* = 0.003) and 0.16 ± 0.09 μm (*p* = 0.000), pre- and postoperatively respectively. In contrast no significant changes were observed in total higher-order and spherical aberrations after surgery (0.34 ± 0.88 μm and 0.06 ± 0.05 μm preoperatively vs. 0.28 ± 0.10 μm and 0.07 ± 0.05 μm postoperatively, respectively; *p* = 0.443 and 0.274).

### Quality of vision questionnaire

**Fig. 2:**
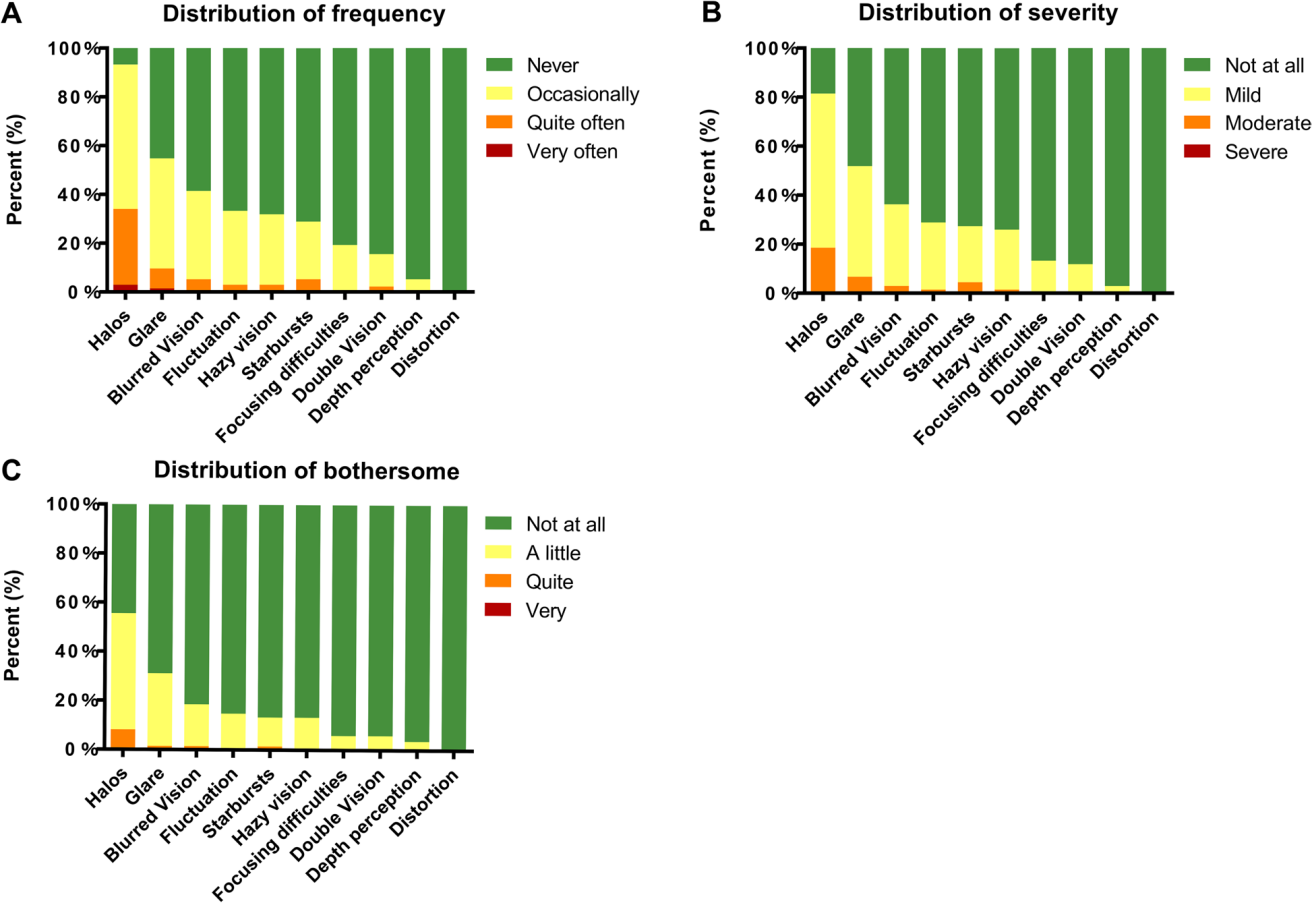
Distribution of frequency, severity, and bothersome of visual symptoms after ICL V4c implantation

At the 6-month follow-up, the most frequent visual symptoms reported by the patients were halos (93.33%), glare (54.81%), blurred vision (41.48%), and fluctuation (33.33%), which were only reported in 8.15%, 1.48%, 1.38%, and 0% of the operated eyes, respectively, as more than mildly bothersome to the patient. The frequency, severity, and bothersome scores of the 10 different postoperative visual symptoms are shown in Fig. 2. The visual symptom frequency, severity, and bothersome scores were 33.87 ± 12.29 (0–59), 25.28 ± 11.29 (0–49), and 15.77 ± 13.31 (0–46), respectively.

### Relationship between tilt, decentration, and visual quality

**Fig. 3:**
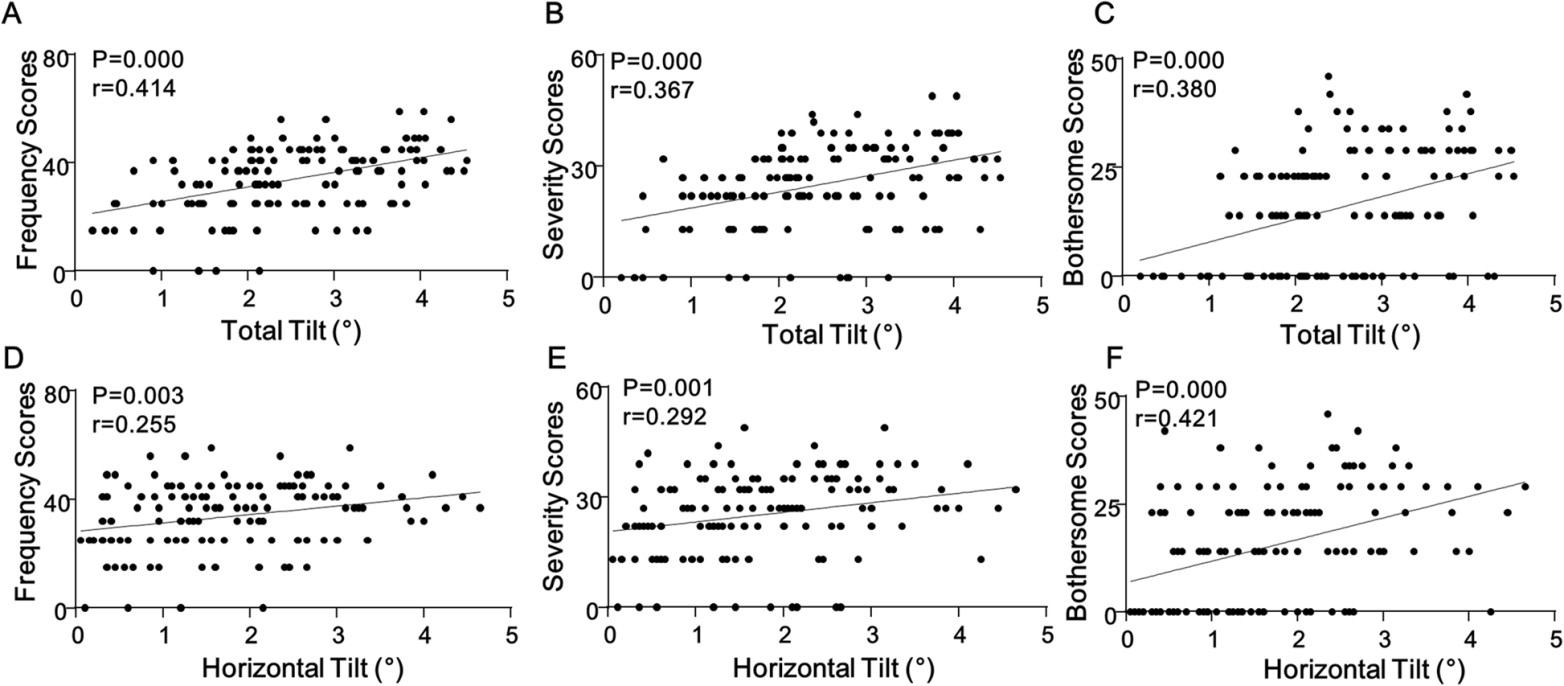
**A-F** Correlation between the total or horizontal tilt and the frequency, severity, or bothersome scores. The X-axis is labeled as total tilt or horizontal tilt, and Y-axis is labeled as the score of frequency, severity and bothersome

Horizontal, vertical, and total ICL tilt or decentration were not significantly correlated with postoperative CDVA, UDVA, higher-order aberration, coma, trefoil, and spherical aberration (*p* > 0.05). Contrastingly, the total and horizontal tilt were positively correlated with frequency (*p* = 0.000, *r* = 0.414, and *p* = 0.003, *r* = 0.255, respectively), severity (*p* = 0.000, *r* = 0.367, and *p* = 0.001, *r* = 0.292, respectively), and bothersome (*p* = 0.000, *r* = 0.380, and *p* = 0.000, *r* = 0.421, respectively) scores of visual symptoms (Fig. 3). No significant correlation was observed between vertical tilt, total decentration, horizontal decentration, vertical decentration, and visual symptom scores.

## Discussion

The clinical efficacy and long-term safety of ICL implantation for myopia correction have been widely recognized [1–5]. However, technological and material developments have led to higher standards of postoperative visual quality in both doctors and patients. Moreover, some patients complained of their visual disturbances despite having visual acuities of 1.0 or better and refractions close to plano after surgeries. Our study is the first to measure and analyze tilt and decentration after ICL V4c implantation and to evaluate their effects on the visual quality of patients.

The CentralFLOW technology in the ICL V4c contains a 360-μm central hole, which provides a reference to fix the center of the ICL and allows observation of ICL decentration relative to the corneal topographic axis. Our study collected images using the AS-OCT, and analyzed the decentration and tilt values of the ICL using MATLAB software. The average total decentration was 0.21 ± 0.12 mm and the 98.5% decentration was within 0.5 mm at 6 months postoperatively. Several clinical studies have reported that the average decentration of IOL post-cataract surgery is approximately 0.23–0.40 mm and the tilt is 3.03°–7.00° [23–25]. Compared with these values, our findings are relatively lower; this discrepancy may be attributed to the location of the ICL haptics on the ciliary sulcus, which allows more stability and less influence from the suspensory ligament and other structures [26]. A study of ICL that used the pupil center as the reference, reported that decentration of 48.9% and 93.6% of eyes were within 0.36 mm and 0.72 mm after ICL implantation, respectively [27]. Notably, the selection criteria of the reference axis remain ununified. In corneal refractive surgery, significant decreases were observed in higher-order aberration and coma, and significant increase in contrast sensitivity when the corneal vertex was used as the correction center rather than the center of the pupil [28–30]. Since it is affected by pupil shape, the pupil axis may not be the best reference to evaluate the tilt and decentration of an ICL [31]. The axis of corneal topography is a line connecting the fixed point of the machine and the first Purkinje image on the anterior surface of the cornea; while the vertex of the cornea is a reflection point on the anterior surface of the cornea [32]. In this study, we used the second generation AS-OCT to measure the tilt and decentration of ICL, which is not affected by the pupil shape and diameter with high repeatability.

A previous study reported that tilt and decentration of the IOL can induce image quality degradation in patients who developed postoperative coma, spherical aberration, and higher-order aberrations [15, 16], especially with an aspheric IOL [33, 34]. Some studies have measured the maximum decentration and tilt of the aspheric IOL, which ensured the comparable visual quality between aspheric and standard IOL. Holladay reported that the critical decentration and tilt values are 0.4 mm and 7°, respectively; visual function will be affected once values are beyond these ranges [33]. The aberration or astigmatism caused by the tilt or decentration of aspheric IOL may affect the image quality of the retina, which inhibits clear image formation of the peripheral part of the retina, leading to glare, halo or monocular diplopia. The spherical shape of the optical zone of the ICL causes the decentration and tilt to have little influence on the objective visual quality. Pérez-Vives [18, 19] reported the results of an ICL decentration simulation test in vitro, and found that coma was directly correlated with the decentration value of the ICL and increased more with pupil diameter and higher refractive degree. Our study showed that tilt and decentration had no significant effect on postoperative aberrations, and had no significant correlation with postoperative UDVA and CDVA after adjusting for age, eye, preoperative refraction, and preoperative scotopic pupil, which is consistent with the results of Park [27]. Additionally, the effect of ICL decentration and tilt on postoperative visual acuity would be clinically insignificant.

The reported visual symptoms of patients after ICL implantation in the order of most to least frequent were halo, glare, blurred vision, fluctuation, hazy vision, starbursts, focusing difficulties, double vision, depth perception, and distortion [14]. Similarly, Chen et al. found that patients with a small pupil diameter had a smaller halo radius after ICL implantation [35]. The largest optical area of ICL V4c was 5.8 mm. In our study, 91.9% of the eyes had scotopic pupil diameter larger than 5.8 mm; therefore, most patients occasionally experienced symptoms such as halo or glare. The total and horizontal tilt were positively correlated with the frequency, severity, and bothersome of visual symptoms scores. Eom et al. suggested that the halo is mainly due to the reflection of the inner wall of the central hole and posterior surface of ICL [13]. Eppig found that light through the central hole has no influence on the postoperative visual quality; however, the off-axis light will increase the intraocular reflection and scattering, which affects the postoperative visual quality [36]. Therefore, visual disturbances may be more evident with severe tilt.

The limitations of this study were an observational study with short follow-up postoperative period and we will design further studies to observe the time-dependent changes of tilt and decentration results post-ICL implantation. Additionally, to overcome these limitations, both eyes were enrolled in this study, and we adjusted for eye laterality, patient age, and preoperative refraction during the statistical analysis to increase the accuracy and robustness of the results. Another limitation of the paper was that the QoV score was a composite of 10 symptoms and it was hard to identify which factor would be the problem. Further study on the effect of tilt or decentration on different visual symptoms after ICL implantation is required.

## Conclusions

ICL V4c implantation can obtain high visual quality and patient satisfaction. Although the degree of tilt and decentration after ICL V4c implantation was small, a positive effect on subjective visual quality was observed.

## Supplementary Information

**Additional file 1.** **Supplementary Figure 1.** A representative raw image from the MATLAB software showing the location of ICL V4c central hole in the eye. The X-axis (horizontal location) and Y-axis (vertical location) are labeled at the bottom and left side of the image, respectively. B. Four registration dotted lines from the top to the bottom are aligned to the anterior (line a) and posterior (line b) surfaces of the cornea and the anterior (line c) and posterior (line d) surfaces of the ICL, respectively. The left and right edge of the ICL central hole are labelled as A and B. For registration, all four dotted lines can be moved (horizontally and vertically), and line c and d can be rotated (clockwise or anti-clockwise) and flexed by clicking relevant buttons in the software. C. The blue dashed line (line e) represents the vertical line passing through the corneal vertex. The center of the central hole is labelled as C, and the decentration of the ICL is determined by calculating the horizontal distance between the point C and the line e on the X-axis. The tilt of the ICL were measured by calculating the average rotation degree of line c and d. **Supplementary Figure 2.** Bland-Altman analysis plot showed consistent results of the ICL tilt (A) and decentration (B) of 135 eyes analyzed by the two examiners.

## Data Availability

The data and Materials are available upon request from the corresponding author at doctzhouxingtao@163.com.

## Abbreviations

ICL: Implantable Collamer Lens
UDVA: Uncorrected distance visual acuity
CDVA: Corrected distance visual acuity
AS-OCT: Anterior segment-optical coherence tomography
ACD: Anterior chamber depth
IOP: Intraocular pressure
ECD: Endothelial cell density
SE: Spherical equivalent
D: Diopters
QoV: Quality of Vision
