# Supplementary material for: Effects of tilt and decentration of Visian Implantable Collamer Lens (ICL V4c) on visual quality: an observational study
Source: BMC Ophthalmol. 2022 Jul 5;22:294. doi: 10.1186/s12886-022-02499-4 (PMC9254425; doi:10.1186/s12886-022-02499-4)
Supplement: Supplementary file 1 — Additional file 1. Supplementary Figure 1. A representative raw image from the MATLAB software showing the location of ICL V4c central hole in the eye. The X-axis (horizontal location) and Y-axis (vertical location) are labeled at the bottom and left side of the image, respectively. B. Four registration dotted lines from the top to the bottom are aligned to the anterior (line a) and posterior (line b) surfaces of the cornea and the anterior (line c) and posterior (line d) surfaces of the ICL, respectively. The left and right edge of the ICL central hole are labelled as A and B. For registration, all four dotted lines can be moved (horizontally and vertically), and line c and d can be rotated (clockwise or anti-clockwise) and flexed by clicking relevant buttons in the software. C. The blue dashed line (line e) represents the vertical line passing through the corneal vertex. The center of the central hole is labelled as C, and the decentration of the ICL is determined by calculating the horizontal distance between the point C and the line e on the X-axis. The tilt of the ICL were measured by calculating the average rotation degree of line c and d. Supplementary Figure 2. Bland-Altman analysis plot showed consistent results of the ICL tilt (A) and decentration (B) of 135 eyes analyzed by the two examiners. [file 12886_2022_2499_MOESM1_ESM.docx]

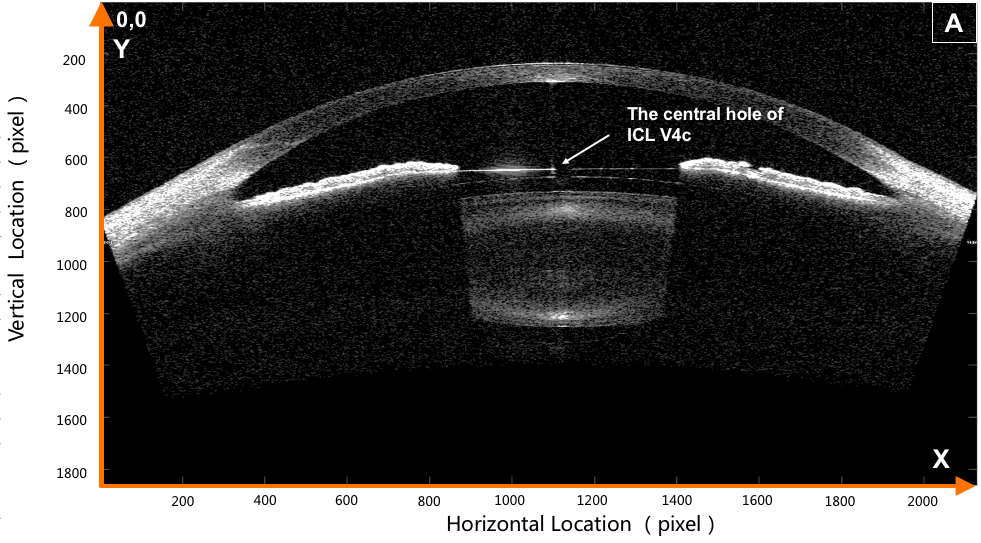


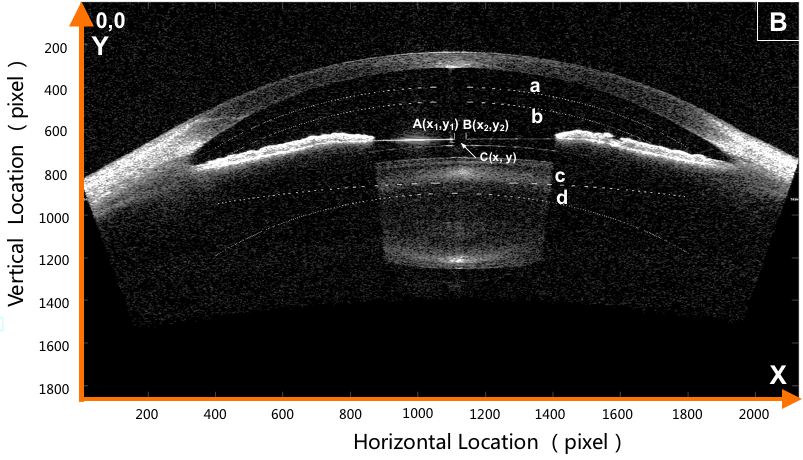


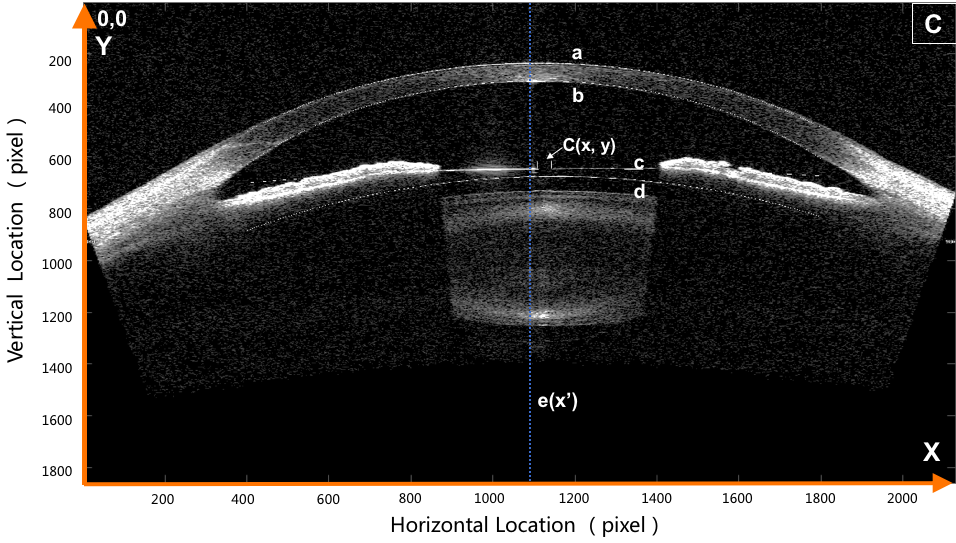


Supplementary Figure 1. A representative raw image from the MATLAB software showing the location of ICL V4c central hole in the eye. The X-axis (horizontal location）and Y-axis（vertical location）are labeled at the bottom and left side of the image, respectively.

B. Four registration dotted lines from the top to the bottom are aligned to the anterior (line a) and posterior (line b) surfaces of the cornea and the anterior (line c) and posterior (line d) surfaces of the ICL, respectively. The left and right edge of the ICL central hole are labelled as A and B. For registration, all four dotted lines can be moved (horizontally and vertically), and line c and d can be rotated (clockwise or anti-clockwise) and flexed by clicking relevant buttons in the software.

C. The blue dashed line (line e) represents the vertical line passing through the corneal vertex. The center of the central hole is labelled as C, and the decentration of the ICL is determined by calculating the horizontal distance between the point C and the line e on the X-axis. The tilt of the ICL were measured by calculating the average rotation degree of line c and d.

Measurements of decentration and tilt of the ICL V4c

After being compensated automatically by anterior segment optical coherence tomography (AS-OCT) for geometric distortions caused by scanning geometry and refraction in the eye, the raw measurement images of AS-OCT were exported to MATLAB software (R2018a, The MathWorks, Inc., Natick, MA, USA) with a purpose-designed program (Supplementary Figure 1A). Four registration lines were manually adjusted to align the anterior and posterior corneal surfaces and the anterior and posterior ICL surfaces. Then the location of marked point on the image were expressed in pixels (x and y) relevant to the coordinate XY axis. By comparing the thickness of cornea vertex from AS-OCT of all 30 eyes of 30 people and their values expressed in pixels from the MATLAB images, per pixel in the MATLAB image equaled to an average of 7.749 μm, and the results were consistent as evaluated by intraclass correlation coefficient (ICC) analysis (P < 0.05, Alpha = 0.956).

The corneal topography axis was defined as the connecting line of the fixation point of the machine and the corneal vertex, which was vertical to the X axis in each image. Before registration, all four registration lines were vertical to the corneal topography axis. For registration, the registration lines (a, b, c and d) were manually moved, and adjusted to fit the anterior and posterior surfaces of the cornea and the ICL in each image, respectively (Supplementary Figure 1B). The tilt value of ICL was determined by averaging the degrees of rotation of the registration lines fitted to the anterior and posterior surfaces of the ICL in each image. To determine the reliability of the data analyzed by the MATLAB software, we analyzed 30 eyes of 30 people consecutively and the tilt values of the lens acquired from the AS-OCT and the MATLAB software were consistent as evaluated by the ICC analysis (P < 0.05, Alpha = 0.911). The highest value of tilt within the 16 images represented the total tilt value. The horizontal and vertical tilt were the values on the 0- and the 90- degree-images.

The left and right edges of the central hole were labelled as point A (x_1_, y_1_) and B (x_2_, y_2_) in each MATLAB image (Supplementary Figure 1B). The center of the central hole, point C (x, y), was used to represent the location of the central hole, and x = (x_1_+x_2_)/2 and y = (y_1_+y_2_)/2. The decentration was defined as the horizontal distance between the central hole and the corneal topographic axis. Since the location of the ICL central hole was not present in all 16 images, the highest value of decentration among the images with a central hole represented the total decentration value. The horizontal and vertical decentration values were based on the total decentration value and calculated according to the image directions (0, 11, 23, 34, 45, 56, 68, 79, 90, 101, 113, 124, 135, 146, 158, and 169 degrees) and the Pythagorean theorem.

In each image of both eyes, the values of registration lines rotated clockwise were positive, and those rotated anti-clockwise were negative. If the center of the central hole was to the right side of the corneal topography axis, the decentration value was positive, otherwise it was negative. Nevertheless, only absolute values were used for the analysis. All image analyses were performed independently by two examiners and the consistency of the results were evaluated. The ICC, Bland-Altman analysis plot, and cross-classification showed consistent results between the two examiners (p < 0.05). All results were averaged from the two analyses.


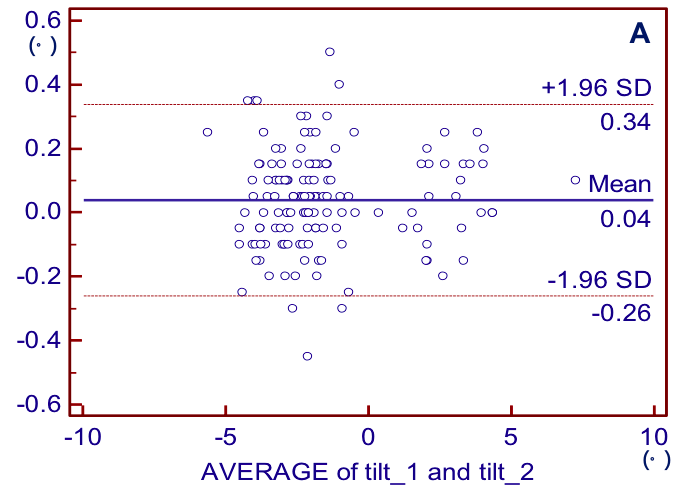


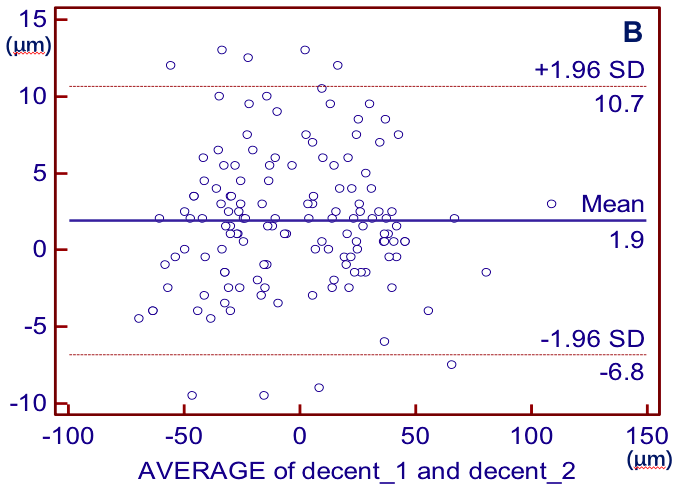


Supplementary Figure 2. Bland-Altman analysis plot showed consistent results of the ICL tilt (A) and decentration (B) of 135 eyes analyzed by the two examiners.
